# Adverse childhood experiences and the risk of non-suicidal self-injury: a meta-analysis

**DOI:** 10.3389/fpsyt.2026.1790171

**Published:** 2026-04-13

**Authors:** Qian He, Yue-jing Wu, Yi-chao Wang

**Affiliations:** Affiliated Mental Health Center & Hangzhou Seventh People’s Hospital, Zhejiang University School of Medicine, Hangzhou, Zhejiang, China

**Keywords:** adverse childhood experiences, meta-analysis, NSSI, non-suicidal self-injury, systematic review

## Abstract

**Background:**

Systematically evaluate the association between Adverse Childhood Experiences (ACEs) and the risk of Non-suicidal Self-Injury (NSSI), thereby providing evidence-based guidance for relevant prevention and early intervention strategies.

**Methods:**

A systematic search was conducted across PubMed, Embase, Web of Science, and the Cochrane Library, from their inception to 30 November 2025, to identify observational studies reporting associations between ACEs and NSSI. Two researchers independently performed literature screening, data extraction, and quality assessment. Effect sizes were pooled using a random-effects model, with association strength expressed as odds ratios (OR) and their 95% confidence intervals (CI). Data analysis was conducted using Stata 15.

**Results:**

A total of 13 articles included. The meta-analysis results suggest that physical abuse [OR = 2.38, 95% CI (1.36, 4.16), I^2^ = 99%], sexual abuse [OR = 1.88, 95% CI (1.24, 2.87), I^2^ = 94.9%], ACEs≥2 [OR = 3.23, 95% CI (2.62, 3.99), I^2^ = 89.9%], ACEs≥3 [OR = 6.13, 95% CI (4.07, 9.24), I^2^ = 96.9%], emotional abuse [OR = 1.65, 95% CI (1.18, 2.32), I^2^ = 97.9%] may increase the risk of NSSI.

**Conclusion:**

In summary, the findings of this meta-analysis suggest that exposure to adverse childhood experiences may be related to an increased likelihood of non-suicidal self-injury. Different forms of childhood adversity, including physical abuse, sexual abuse, and emotional abuse, as well as cumulative exposure to multiple ACEs, were associated with higher risks of NSSI.

**Systematic review registration:**

https://www.crd.york.ac.uk/prospero/, identifier CRD42026128495.

## Background

Adverse childhood experiences (ACEs) refer to a range of stressful or potentially traumatic events occurring before the age of 18 that may disrupt normal psychological and social development (1). In epidemiological research, ACEs are commonly conceptualized within three domains: abuse (including physical, emotional, or sexual abuse), neglect (physical or emotional neglect), and household dysfunction, such as parental mental illness, substance abuse, domestic violence, incarceration of a family member, or parental separation (2, 3). Exposure to these early-life adversities has been widely recognized as an important determinant of long-term health outcomes.

Accumulating evidence suggests that ACE exposure is associated with a variety of adverse mental health outcomes, including depression, anxiety disorders, substance misuse, and suicidal behaviors (4). In addition to psychological consequences, early-life stress may also influence long-term physical health. Previous studies indicate that chronic exposure to childhood adversity can affect biological systems involved in stress regulation, including the neuroendocrine and immune systems, thereby increasing the risk of chronic diseases such as cardiovascular and metabolic disorders later in life (5, 6).

Non-suicidal self-injury (NSSI) refers to the deliberate infliction of physical harm upon oneself without the intent to die (7). The behavior is most frequently observed among adolescents and young adults and is often associated with emotional distress, interpersonal difficulties, and maladaptive coping strategies (8, 9). In recent years, NSSI has attracted increasing attention as a public health concern due to its relatively high prevalence among young people and its potential role as a precursor to suicidal behavior (10). Early identification and intervention are therefore considered important for reducing subsequent suicide risk (11).

A growing number of studies have explored the relationship between ACEs and NSSI. Several theoretical perspectives suggest that exposure to childhood adversity may impair the development of effective emotion regulation abilities, leaving individuals more vulnerable to intense emotional distress. Under such circumstances, self-injury may function as a maladaptive strategy to regulate overwhelming emotions or psychological pain (12, 13). In addition, experiences such as emotional neglect, family conflict, or unstable household environments may weaken social support and coping resources, further increasing vulnerability to self-harm behaviors (14). Biological pathways may also play a role. Early-life stress has been shown to influence brain development and stress-response systems, which may contribute to increased susceptibility to self-injurious behavior (15).

Although numerous studies have investigated the association between ACE exposure and NSSI, the reported findings remain inconsistent across populations and study designs. Differences in ACE definitions, measurement methods, and participant characteristics may partly explain these variations. To date, a comprehensive quantitative synthesis evaluating the overall relationship between ACE exposure and NSSI risk remains limited (16). Therefore, the present study aimed to systematically review the existing literature and conduct a meta-analysis to quantify the association between adverse childhood experiences and non-suicidal self-injury. By integrating available evidence, this study seeks to provide a clearer understanding of the relationship between early-life adversity and self-injurious behaviors and to inform prevention strategies for vulnerable populations.

## Methods

This systematic evaluation and meta-analysis will strictly follow the PRISMA (Preferred Reporting Items for Systematic Reviews and Meta-Analyses) guidelines (17). Register with Prospero, with the registration number CRD42026128495.

### Literature retrieval

A comprehensive literature search was performed in four electronic databases, including PubMed, Embase, the Cochrane Library, and Web of Science, from database inception to 30 November 2025. The search strategy combined Medical Subject Headings (MeSH) and free-text terms related to adverse childhood experiences and non-suicidal self-injury. The main search terms included “Adverse Childhood Experiences,” “childhood adversity,” “childhood trauma,” “ACEs,” “non-suicidal self-injury,” “self-harm,” and “deliberate self-harm.” Boolean operators (AND/OR) were used to combine search terms appropriately. In addition, the reference lists of relevant reviews and included studies were manually screened to identify additional eligible articles that may not have been captured in the database search.

### Inclusion and exclusion criteria

#### Inclusion criteria

1. Study Type: Prospective or retrospective observational studies (cohort studies and Cross-sectional study).
2. Study Population: The study subjects (age<18years) are individuals who have experienced ACEs and can provide relevant data on the relationship between ACEs and NSSI. There are no age restrictions for participants.
3. Assessment tools: Valid standardized instruments or scales must be employed in the study to evaluate ACEs and NSSI, including: the Adverse Childhood Experiences Scale; assessment tools for non-suicidal self-injury (Self-Injury Inventory, Deliberate Self-Harm Inventory).
4. The study must provide quantitative data (adjusted odds ratios (OR)) and their 95% confidence intervals regarding the relationship between exposure to ACEs and the occurrence of NSSI.

#### Exclusion criteria

1. Exclude articles that do not conform to the observational study category, such as experimental studies, clinical trials, and drug intervention studies.
2. The study did not provide sufficient data on the relationship between ACEs exposure and NSSI, or it was not possible to extract relevant data for statistical analysis.
3. If multiple publications were based on the same dataset or study population, only one study was included to avoid duplicate data; in such cases, the article with the most comprehensive information or the largest sample size was retained.
4. Studies where full-text access is unavailable.

### Study selection

During the literature screening process, two researchers independently used EndNote 21 software to initially screen the literature obtained from the search, first through the titles and abstracts, and then to exclude literature that clearly did not meet the inclusion criteria. Subsequently, the remaining literature was reviewed by reading the full text in its entirety to further determine whether it met the inclusion and exclusion criteria. In case of disagreement between the two researchers during the screening process, it would be resolved through discussion and negotiation; if the negotiation still failed to reach a consensus, a third researcher would be invited to adjudicate to ensure the objectivity and consistency of the screening process.

### Data extractions

The extraction included the basic information of the study (first author, year of publication, country, and study design), characteristics of the study population (sample size, gender distribution, mean age, and type of ACEs), and effect estimates describing the association between ACE exposure and NSSI. When studies reported both crude and adjusted odds ratios, the most fully adjusted estimates were extracted whenever available. If adjusted estimates were not reported, crude odds ratios were used. In the process of data extraction, if two investigators disagreed on the data, it would be resolved through negotiation, and if no agreement could be reached, a third investigator would adjudicate to ensure the accuracy and consistency of data extraction.

### Assessment of risk of bias

The risk of bias in the included studies will be evaluated independently by two investigators, and the results will be cross-checked. For cohort and case-control studies, the Newcastle Ottawa Scale (NOS (18)) will be used to assess quality. The NOS evaluates studies based on three dimensions: population selection, comparability, and exposure or outcome, with eight items totaling nine points. Scores range from 0 to 4 (low quality), 5 to 6 (moderate quality), and 7 to 9 (high quality). Studies scoring 0 to 4 will be excluded. For cross-sectional studies, this study will use the AHRQ quality (19) assessment tool to evaluate quality. This tool primarily assesses the rationality of the study design, the representativeness of the sample selection, the clarity of the definitions of exposure and outcome, the accuracy of the data collection process, the rationality of the statistical methods, and the completeness of the report.

### Statistical analysis

In this study, the random effects model was adopted due to the high degree of heterogeneity among the included studies. The risk ratio (OR) and corresponding 95% confidence interval (CI) were extracted for each study and then pooled together. Statistical heterogeneity among studies was evaluated using the I² statistic. Values of 25%, 50%, and 75% were considered to represent low, moderate, and high heterogeneity, respectively. To detect publication bias, a funnel plot was generated, and its asymmetry was examined. If the funnel plot showed signs of asymmetry, Egger’s test was performed to assess the statistical significance of the bias. A p-value of < 0.05 suggests the presence of publication bias, while a p-value of > 0.05 indicates no significant bias. If necessary, the trim-and-fill method may be applied to adjust for any potential publication bias and verify the robustness of the results. To further explore potential sources of between-study heterogeneity, meta-regression analyses were conducted for outcomes with substantial heterogeneity. The following study-level variables were examined as potential moderators: mean age of participants, publication year, country, study design, and type of adverse childhood experiences. These analyses were performed to assess whether study characteristics contributed to variations in the pooled effect estimates.

## Results

### Literature search results

**Figure 1:**
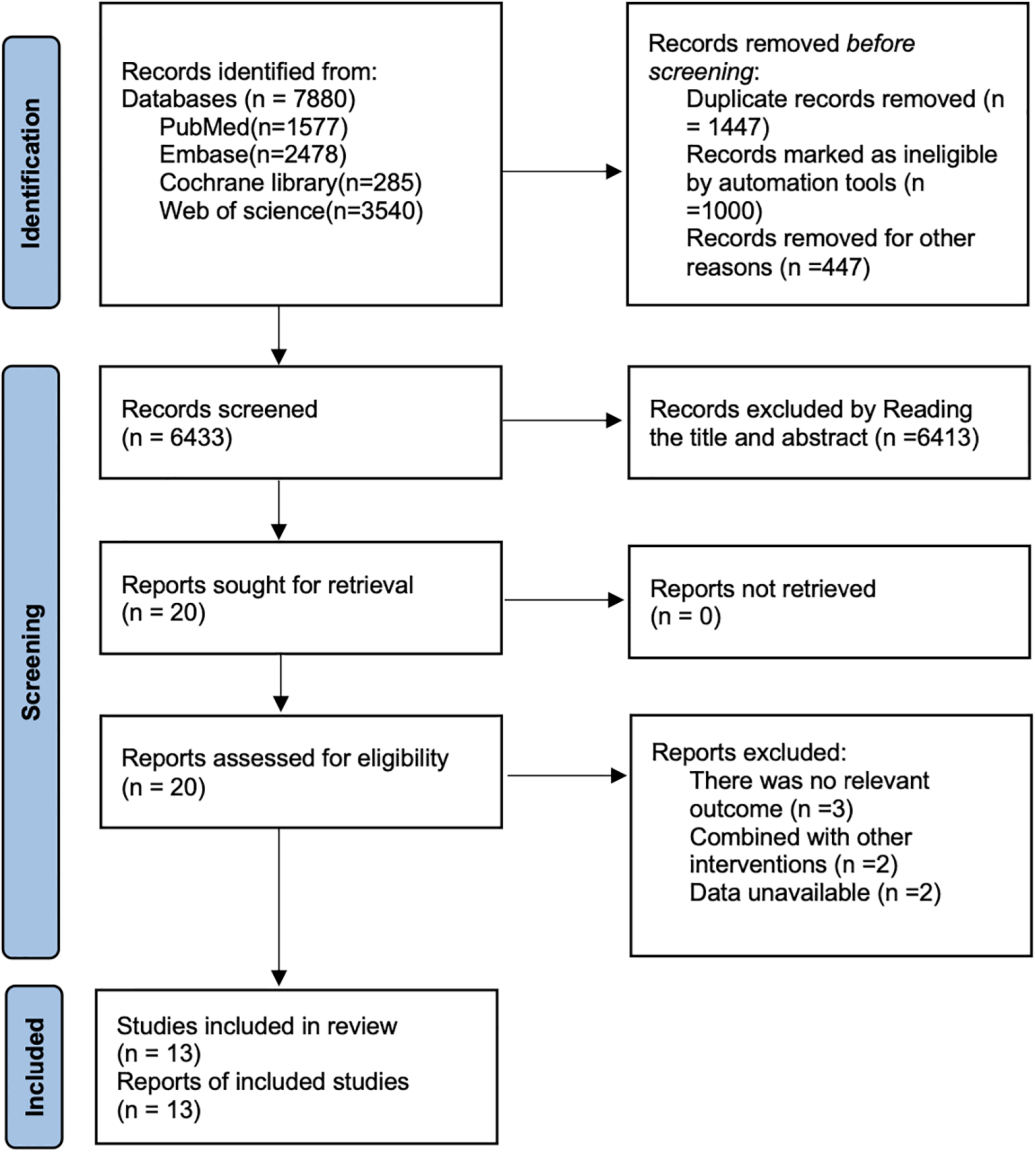
Literature search flow chart.

As illustrated in **Figure 1**, a total of 7,880 articles were retrieved through searches of PubMed (n=1,577), Embase (n=2,478), the Cochrane Library (n=285), and Web of Science (n=3,540). After removing 1,447 duplicate records, 6,413 articles were excluded based on title and abstract screening, and a further 7 articles were excluded following full-text review. Ultimately, 13 articles (20–32) were included for analysis.

### Basic characteristics of included literature

A total of 13 studies were included in this meta-analysis. Most studies were conducted in China (n = 8), with the remaining studies carried out in Canada, the United States, Finland, Germany, and Sweden. In terms of study design, the majority were cross-sectional studies (n = 12), while one study adopted a cohort design. Sample sizes varied considerably across studies, ranging from 125 to 32,310,774 participants. The participants were primarily adolescents, with mean ages ranging from 12.0 to 17.8 years, and the gender distribution was generally balanced. Most studies examined multiple forms of adverse childhood experiences, particularly physical abuse, emotional abuse, and sexual abuse, while several studies also included broader adversities such as household substance abuse, exposure to community or domestic violence, sex discrimination, and bullying. All included studies assessed the association between ACE exposure and non-suicidal self-injury using logistic regression models. Detailed characteristics of the included studies are summarized in **Table 1**.

**Table 1:** Table of basic characteristics of included literature.

Study	Year	Study design	Country	Sample size	Gender (M/F)	Mean age(years)	Type of adverse childhood experiences	Regression model
Baiden	2017 (20)	cross-sectional	Canada	2038	1246/792	12.4	Emotional abuse; Physical abuse; Sexual abuse	logistic regression
Carbone	2021 (21)	cross-sectional	USA	32,310,774	16526960/15783814	12	Separation or divorce; Death of a family member; physical abuse; emotional abuse	logistic regression
Fan	2025 (22)	cross-sectional	China	152	43/109	15.43	Emotional abuse; Physical abuse; Sexual abuse	logistic regression
He	2024 (23)	cross-sectional	China	95549	47617/47932	14.9	physical abuse, emotional abuse, sexual abuse, household substance abuse, witness of community violence, witness of domestic violence, sex discrimination, and bullying	logistic regression
Isohookana	2013 (24)	cohort study	Finland	508	208/300	15.45	Emotional abuse; Physical abuse; Sexual abuse	logistic regression
Kaess	2013 (25)	cross-sectional	Germany	125	62/63	17.1	Emotional abuse; Physical abuse; Sexual abuse	logistic regression
Laporte	2023 (26)	cross-sectional	Sweden	184	100/84	17.8	physical abuse, emotional abuse, sexual abuse, household substance abuse, witness of community violence, witness of domestic violence, sex discrimination, and bullying	logistic regression
Li	2019 (28)	cross-sectional	China	1810	1005/905	17	Emotional abuse; Physical abuse; Sexual abuse	logistic regression
Li	2022 (27)	cross-sectional	China	14500	7347/7153	14.38	physical abuse, emotional abuse, sexual abuse, household substance abuse, witness of community violence, witness of domestic violence, sex discrimination, and bullying	logistic regression
Wan	2019 (29)	cross-sectional	China	15278	8278/7000	15.44	Emotional abuse; Physical abuse; Sexual abuse	logistic regression
Wang	2024 (30)	cross-sectional	China	562	216/346	16.76	Emotional abuse; Physical abuse; Sexual abuse	logistic regression
Xiao	2023 (31)	cross-sectional	China	16853	8853/8000	14.7	Emotional abuse; Physical abuse; Sexual abuse	logistic regression
Zhang	2023 (32)	cross-sectional	China	18723	10000/8723	17	Emotional abuse; Physical abuse; Sexual abuse	logistic regression

### Risk of bias results

The quality assessment scores for this study are presented in **Table 2**. Cohort studies scored 8–9 points, The methodological quality of cross-sectional studies was assessed using the AHRQ checklist (11 items). The total scores ranged from 7 to 8, indicating moderate to high methodological quality among the included studies.

**Table 2:** Risk of bias results.

Cross-sectional																					
Study	Whether the source of the information is clear		Whether exposed and non-exposed groups are listed		Whether a time was given to identify patients	If not, population derived, whether the subjects were consecutive		Whether the subjective factors of the evaluator cover up other aspects of the research object		Any assessment performed to ensure quality is described	The rationale for excluding any patients from the analysis was explained		Describe measures to evaluate and/or control for confounding factors		explain how missing data were handled in the analysis		Response rates and the completeness of data collection are summarized		If there is follow-up, identify the percentage of patients with expected incomplete data or follow-up results		Total scores
Baiden 2017 (20)	Yes		Unclear		Yes	Yes		Yes		Yes	Yes		Yes		Yes		Unclear		Yes		8
Carbone 2021 (21)	Yes		Unclear		Unclear	Unclear		Yes		Yes	Yes		Yes		Yes		Unclear		Yes		7
Fan 2025 (22)	Yes		Unclear		Yes	Yes		Yes		Yes	Yes		Yes		Yes		Unclear		Yes		8
He 2024 (23)	Yes		Unclear		Yes	Yes		Yes		Yes	Yes		Yes		Yes		Unclear		Yes		8
Kaess 2013 (25)	Yes		Unclear		Yes	Yes		Yes		Yes	Yes		Yes		Yes		Unclear		Yes		8
Laporte 2023 (26)	Yes		Unclear		Yes	Yes		Yes		Yes	Yes		Yes		Yes		Unclear		Yes		8
Li 2019 (28)	Yes		Unclear		Unclear	Unclear		Yes		Yes	Yes		Yes		Yes		Unclear		Yes		7
Li 2022 (27)	Yes		Unclear		Unclear	Unclear		Yes		Yes	Yes		Yes		Yes		Unclear		Yes		7
Wan 2019 (29)	Yes		Unclear		Unclear	Unclear		Yes		Yes	Yes		Yes		Yes		Unclear		Yes		7
Wang 2024 (30)	Yes		Unclear		Yes	Yes		Yes		Yes	Yes		Yes		Yes		Unclear		Yes		8
Xiao 2023 (31)	Yes		Unclear		Unclear	Unclear		Yes		Yes	Yes		Yes		Yes		Unclear		Yes		7
Zhang 2023 (32)	Yes		Unclear		Unclear	Unclear		Yes		Yes	Yes		Yes		Yes		Unclear		Yes		7
Cohort study																					
Study		Representativeness of the exposed group		Selection of non-exposed groups			Determination of exposure factors		Identification of outcome indicators not yet to be observed at study entry			Comparability of exposed and unexposed groups considered in design and statistical analysis		design and statistical analysis		Adequacy of the study’s evaluation of the outcome		Adequacy of follow-up in exposed and unexposed groups		Total scores	
Isohookana 2013 (24)		*		*			*		/			**		*		*		*		8	

* means one scores.

** means two scores.

### Meta-analysis results

#### Physical abuse

**Figure 2:**
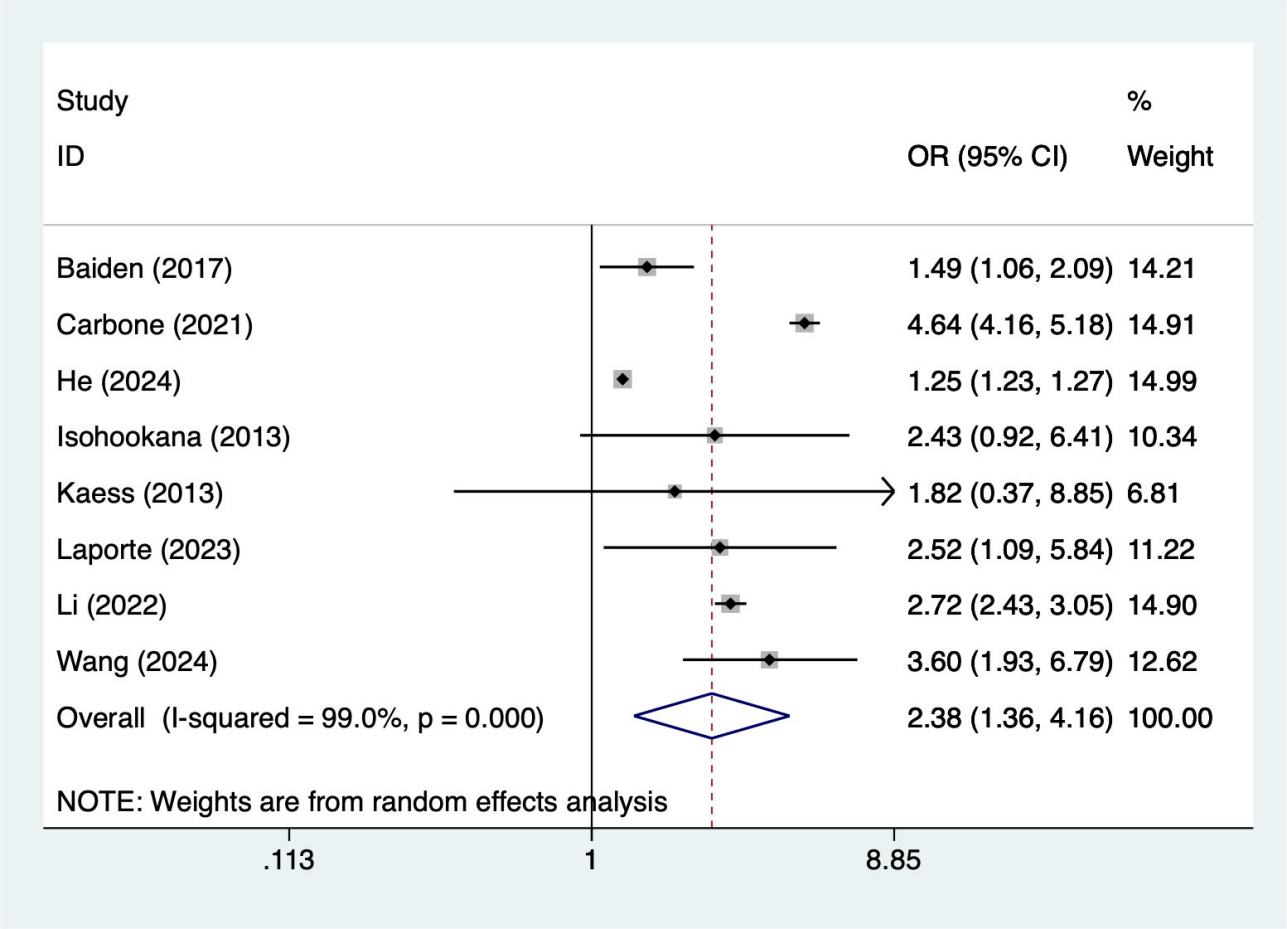
Forest plot of meta-analysis of physical abuse.

8 studies reported physical abuse. Heterogeneity testing (I² = 99%, P = 0.001) was conducted using a random-effects model. The analysis results (**Figure 2**) suggest that physical abuse may increase the risk of NSSI [OR = 2.38, 95% CI (1.36, 4.16)]. Sensitivity analysis results (**Supplementary Material Figure S1**) indicate this finding is relatively robust and not influenced by any single study.

#### Sexual abuse

**Figure 3:**
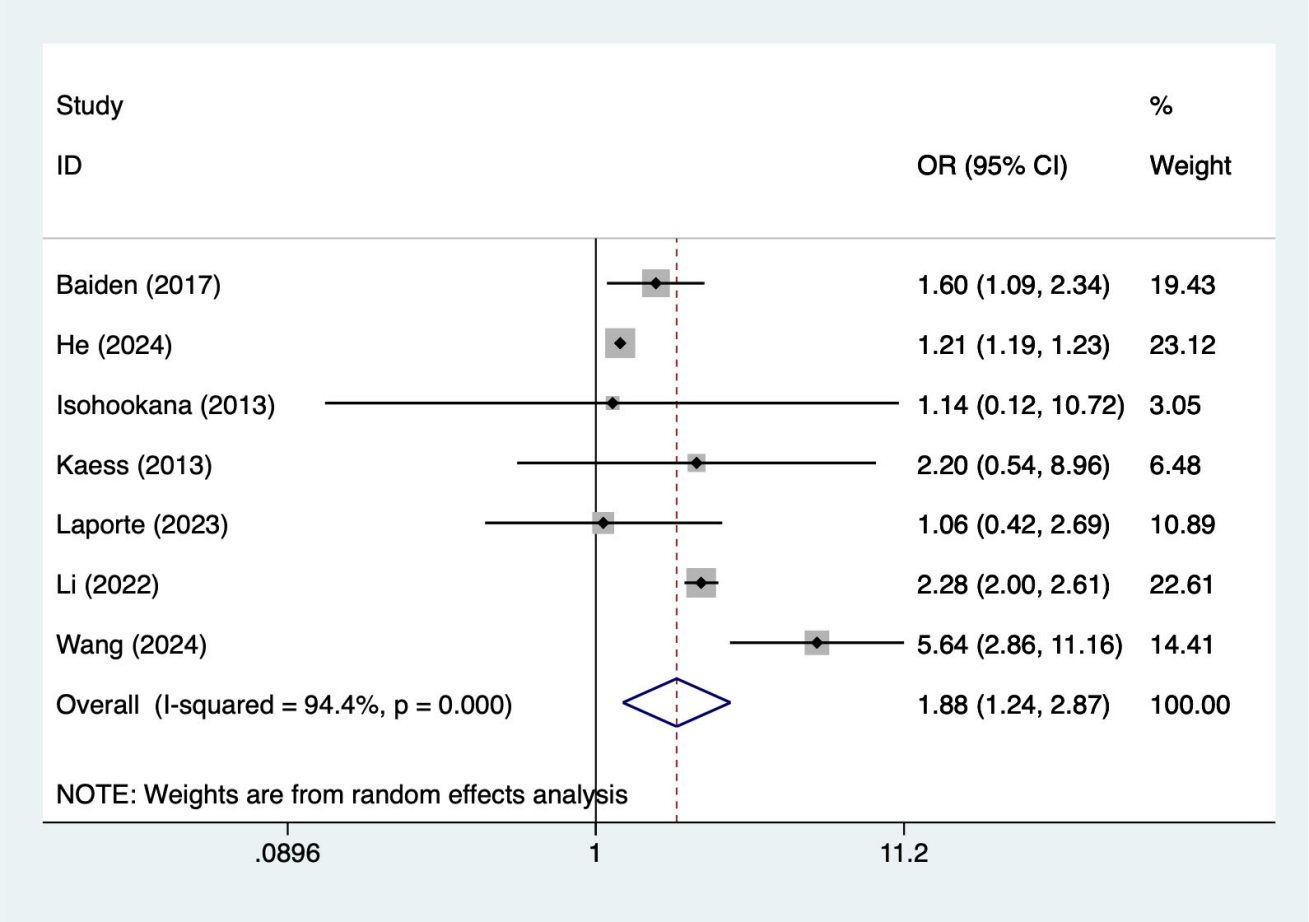
Forest plot of meta-analysis of sexual abuse.

7 studies reported sexual abuse. Heterogeneity testing (I² = 94.4%, P = 0.001) was conducted using a random-effects model. The analysis results (**Figure 3**) suggest that sexual abuse may increase the risk of NSSI [OR = 1.88, 95% CI (1.24, 2.87)]. Sensitivity analysis results (**Supplementary Material Figure S2**) indicate this finding is relatively robust and not influenced by any single study.

#### ACEs≥2

**Figure 4:**
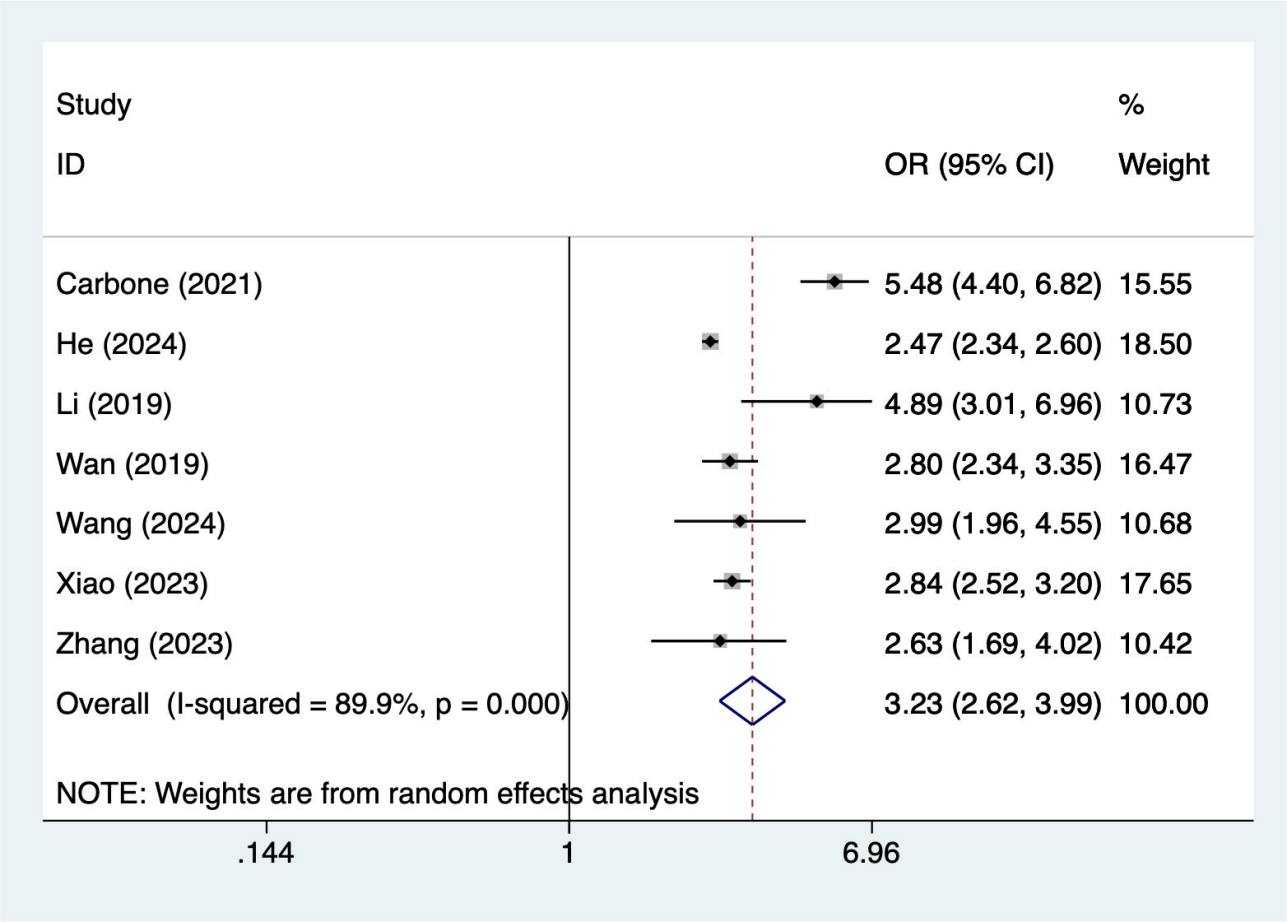
Forest plot of meta-analysis of ACEs≥2.

7 studies reported ACEs≥2. Heterogeneity testing (I² = 89.9%, P = 0.001) was conducted using a random-effects model. The analysis results (**Figure 4**) suggest that ACEs≥2 may increase the risk of NSSI [OR = 3.23, 95% CI (2.62, 3.99)]. Sensitivity analysis results (**Supplementary Material Figure S3**) indicate this finding is relatively robust and not influenced by any single study.

#### ACEs≥3

**Figure 5:**
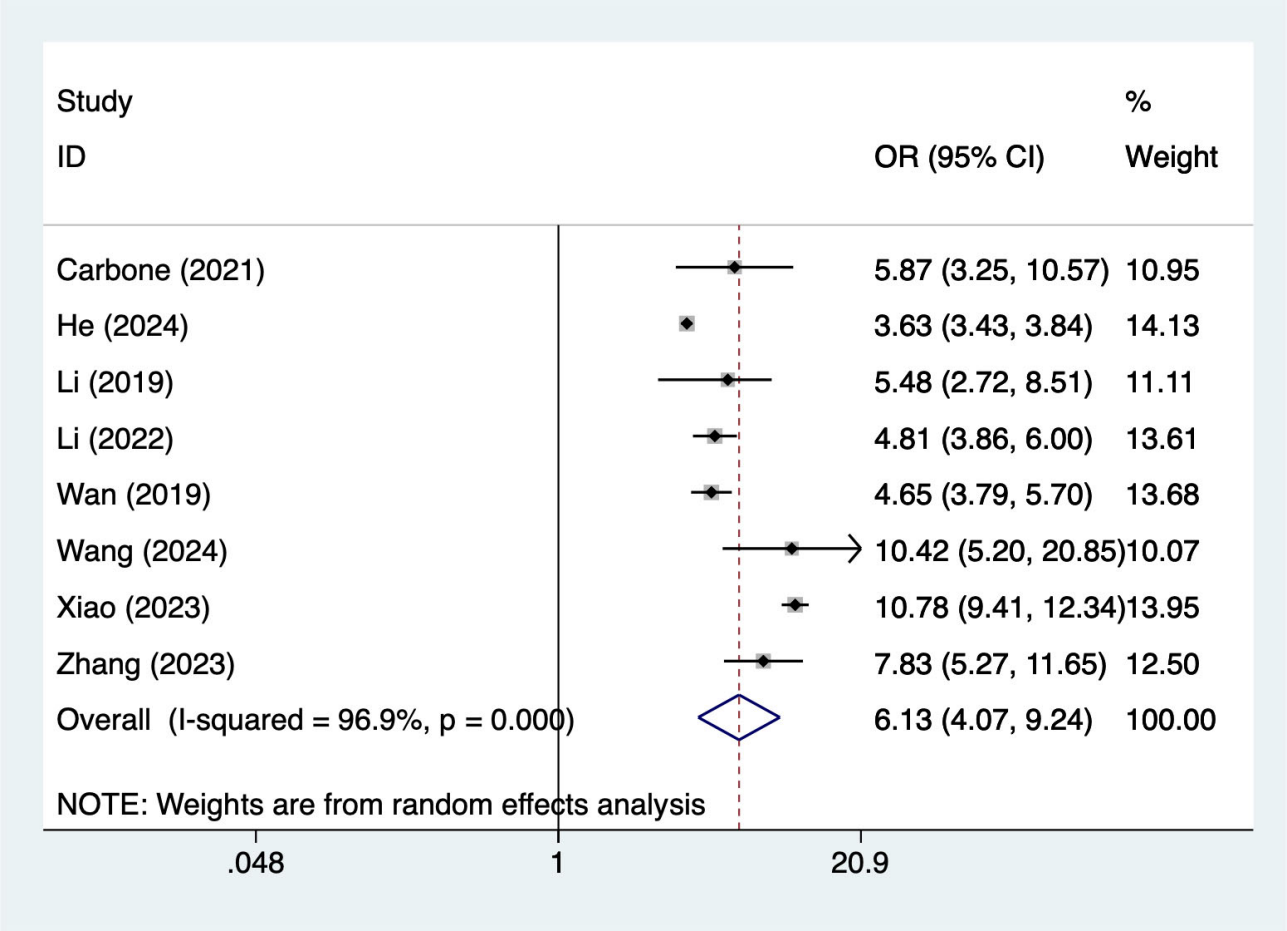
Forest plot of meta-analysis of ACEs≥3.

8 studies reported ACEs≥3. Heterogeneity testing (I² = 96.9%, P = 0.001) was conducted using a random-effects model. The analysis results (**Figure 5**) suggest that ACEs≥3 may increase the risk of NSSI [OR = 6.13, 95% CI (4.07, 9.24)]. Sensitivity analysis results (**Supplementary Material Figure S4**) indicate this finding is relatively robust and not influenced by any single study.

#### Emotional abuse

**Figure 6:**
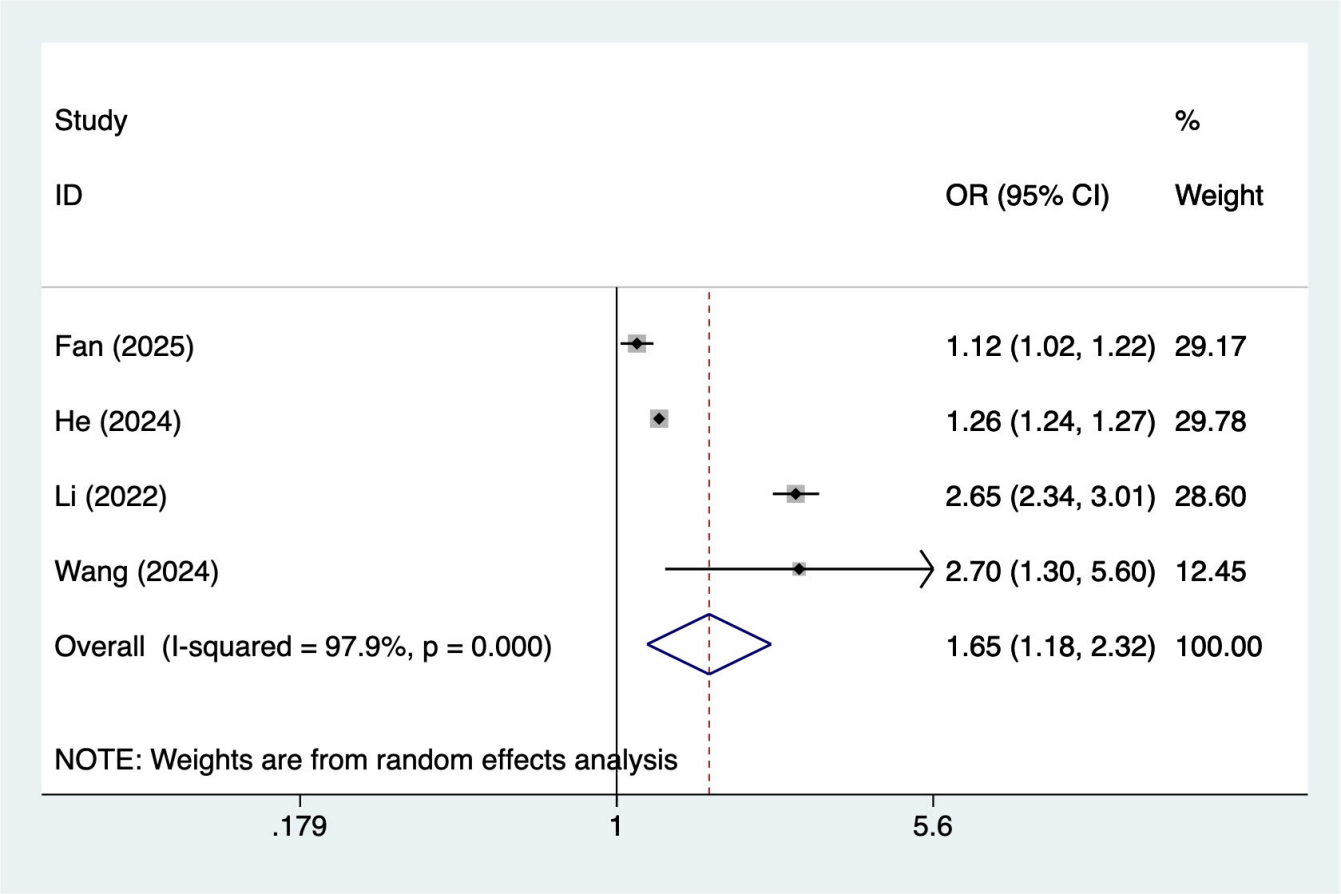
Forest plot of meta-analysis of emotional abuse.

4 studies reported emotional abuse. Heterogeneity testing (I² = 97.9%, P = 0.001) was conducted using a random-effects model. The analysis results (**Figure 6**) suggest that emotional abuse may increase the risk of NSSI [OR = 1.65, 95% CI (1.18, 2.32)]. Sensitivity analysis results (**Supplementary Material Figure S5**) indicate this finding is relatively robust and not influenced by any single study. Because the number of included studies was relatively small, the statistical power of this analysis may be limited.

### Meta-regression results

To further explore potential sources of heterogeneity, meta-regression analyses were conducted using study-level covariates including mean age, publication year, country, study design, and type of adverse childhood experiences (ACEs).

The results (**Supplementary Material Table S2**) indicated that type of ACEs was significantly associated with heterogeneity in the physical abuse analysis (β = 0.21, P = 0.01). However, no significant associations were observed for mean age, publication year, country, or study design.

For the other outcomes, including sexual abuse, ACEs ≥2, ACEs ≥3, and emotional abuse, none of the examined covariates significantly explained the observed heterogeneity (all P > 0.05). These findings suggest that differences in ACE type may partly contribute to heterogeneity in studies examining physical abuse, whereas other study-level factors did not show significant moderating effects. Because the number of studies included in each outcome analysis was relatively small, subgroup analyses by country or age were not performed.

### Publication bias

Publication bias was assessed using funnel plots and Egger’s test. However, because several analyses included a relatively small number of studies, the results of these tests should be interpreted with caution. Funnel plot results (**Supplementary Materials Figures S6**–**S10**) indicated no significant publication bias for Physical abuse (P = 0.145), Sexual abuse (P = 0.174), ACEs≥2 (P = 0.101), ACEs ≥3 (P = 0.208), and emotional abuse (P = 0.471) indicated a low likelihood of publication bias for these indicators.

## Discussion

The present study synthesized available observational evidence to examine the association between ACEs and NSSI. Overall, the pooled estimates suggest that exposure to several forms of childhood adversity may be associated with a higher likelihood of NSSI. Experiences such as physical abuse, sexual abuse, and emotional abuse were consistently associated with higher odds of self-injurious behaviors. In addition, analyses examining cumulative exposure indicated that individuals reporting multiple ACEs (ACEs ≥ 2 or ACEs ≥ 3) tended to show higher odds of engaging in NSSI. These findings are broadly consistent with previous research exploring the psychological consequences of early adverse environments.

Among the specific types of ACEs examined, physical abuse showed a relatively strong association with NSSI. Individuals who reported experiences of physical abuse appeared more likely to engage in self-injurious behaviors than those without such experiences. Although the level of heterogeneity across studies was substantial (I² = 99%), sensitivity analyses indicated that the overall effect estimate remained relatively stable when individual studies were removed. Previous studies have suggested that childhood physical abuse may be linked to emotional dysregulation, impulsivity, and negative self-perceptions (33, 34). These psychological characteristics are frequently reported in individuals who engage in NSSI and may help explain why early experiences of violence are related to later self-injurious behaviors.

A comparable pattern was observed for sexual abuse. The pooled results indicated a higher likelihood of NSSI among individuals with a history of sexual abuse (35). Although heterogeneity across studies remained high (I² = 94.4%), the direction of the association was relatively consistent. Earlier research has shown that sexual abuse may be associated with a range of psychological consequences, including shame, post-traumatic stress symptoms, and difficulties in interpersonal relationships (36, 37). These factors may be associated with a greater likelihood of maladaptive coping behaviors, including self-injury.

Emotional abuse also showed a positive association with NSSI. However, the number of studies included in this analysis was relatively small, which may limit the statistical power and stability of the pooled estimate. Emotional abuse may occur through persistent criticism, rejection, or emotional neglect, and its association with psychological distress may persist over time (38). Compared with physical or sexual abuse, emotional abuse is sometimes less visible, but its psychological impact may be equally significant. The pooled results suggested a moderate association between emotional abuse and NSSI, and sensitivity analyses indicated that the findings were relatively stable (39). These observations highlight the importance of considering emotional forms of childhood adversity when examining the risk factors for self-injurious behavior.

In addition to individual ACE categories, the present analysis also considered the cumulative impact of multiple adverse experiences. The results suggested a gradient association, with higher numbers of ACE exposures corresponding to greater odds of NSSI. Individuals who reported three or more ACEs showed particularly elevated odds of self-injury (40, 41). This pattern is consistent with the cumulative risk framework, which suggests that multiple adverse experiences may be jointly associated with more pronounced difficulties in psychological functioning and behavioral outcomes (42). It should also be noted that considerable heterogeneity was observed across most analyses. Several factors may contribute to this variability. The included studies differed in terms of study populations, geographical locations, and cultural contexts. In addition, the instruments used to assess ACE exposure and NSSI varied across studies, which may influence reported associations. Differences in study design and adjustment for confounding variables may also partly explain the observed heterogeneity. Given these differences, the pooled estimates should be interpreted cautiously.

### Strengths and limitations

This study brings together findings from multiple observational studies examining the relationship between adverse childhood experiences and non-suicidal self-injury. By combining results across different populations and settings, the meta-analysis provides a broader perspective on how early adverse experiences may relate to self-injurious behaviors. In addition, both individual ACE categories and cumulative exposure levels were considered, which helps illustrate how different forms of adversity may be associated with NSSI risk.

Nevertheless, several limitations should be acknowledged. Most of the included studies were cross-sectional in design. As cross-sectional studies measure exposure and outcomes at the same time point, it is not possible to determine temporal relationships or infer causality. Therefore, the findings of this meta-analysis should be interpreted as associations rather than causal effects. In addition, substantial heterogeneity was present in several pooled analyses. Differences in population characteristics, measurement approaches, and cultural contexts may partly account for this variability. Although sensitivity analyses suggested that the pooled estimates were relatively stable, the magnitude of the associations should still be interpreted with caution. Furthermore, some analyses were based on a limited number of studies, which may reduce the statistical power of subgroup analyses and affect the reliability of publication bias assessments. Finally, residual confounding cannot be completely ruled out because not all original studies adjusted for the same set of potential confounders.

### Practical implications

From a practical perspective, these findings highlight the importance of early identification of adolescents who have experienced adverse childhood experiences. Screening for ACE exposure in school settings or youth health services may help identify individuals who are at increased risk of engaging in self-injurious behaviors. Early identification could allow timely psychological support and preventive interventions. In addition, school-based prevention programs may play an important role in reducing the potential impact of childhood adversity. Interventions that focus on improving emotional regulation skills, strengthening coping strategies, and enhancing social support networks may be particularly beneficial for adolescents who have experienced adverse environments. Creating supportive school environments and increasing awareness among teachers and counselors may also help facilitate early intervention and support adolescents who may be at elevated risk of self-injurious behaviors.

## Conclusion

In summary, the findings of this meta-analysis suggest that exposure to adverse childhood experiences may be related to an increased likelihood of non-suicidal self-injury. Different forms of childhood adversity, including physical abuse, sexual abuse, and emotional abuse, as well as cumulative exposure to multiple ACEs, were associated with higher risks of NSSI. However, given the substantial heterogeneity observed across studies, these findings should be interpreted with caution. Future longitudinal studies are needed to further clarify the temporal relationship and potential mechanisms underlying the association between childhood adversity and self-injurious behaviors.

## Data Availability

The original contributions presented in the study are included in the article/**Supplementary Material**. Further inquiries can be directed to the corresponding author.
