# Supplementary material for: Adverse childhood experiences and the risk of non-suicidal self-injury: a meta-analysis
Source: Front Psychiatry. 2026 Apr 13;17:1790171. doi: 10.3389/fpsyt.2026.1790171 (PMC13111225; doi:10.3389/fpsyt.2026.1790171)
Supplement: Supplementary file 1 [file DataSheet1.docx]

Table s1 search history

(("Adverse Childhood Experiences"[Mesh] OR "adverse childhood experience*"[Title/Abstract] OR "childhood adversit*"[Title/Abstract] OR "childhood trauma"[Title/Abstract] OR "early life stress"[Title/Abstract] OR "childhood maltreatment"[Title/Abstract]) AND ("Self-Injurious Behavior"[Mesh] OR "non-suicidal self-injury"[Title/Abstract] OR "nonsuicidal self injury"[Title/Abstract] OR "self harm"[Title/Abstract] OR "self-harm"[Title/Abstract] OR "deliberate self harm"[Title/Abstract] OR "self injur*"[Title/Abstract])).

Table S2 Meta-regression analysis results

| Covariate | Physical abuse (β, P value) | Sexual abuse (β, P value) | ACEs ≥2 (β, P value) | ACEs ≥3 (β, P value) | Emotional abuse (β, P value) |
| --- | --- | --- | --- | --- | --- |
| Mean age | 0.65, 0.12 | 0.11, 0.26 | 0.45, 0.23 | 0.26, 0.77 | 0.51, 0.27 |
| Publication year | -0.26, 0.65 | 0.87, 0.17 | 0.33, 0.75 | 0.56, 0.45 | 0.37, 0.24 |
| Country | 0.26, 0.17 | 0.91, 0.54 | 0.23, 0.26 | 0.34, 0.75 | 0.43, 0.81 |
| Study design | 0.86, 0.41 | -0.27, 0.13 | 0.11, 0.37 | 0.56, 0.34 | 0.36, 0.28 |
| Type of ACEs | 0.21, 0.01 | 0.37, 0.19 | 0.34, 0.47 | 0.27, 0.44 | 0.19, 0.34 |


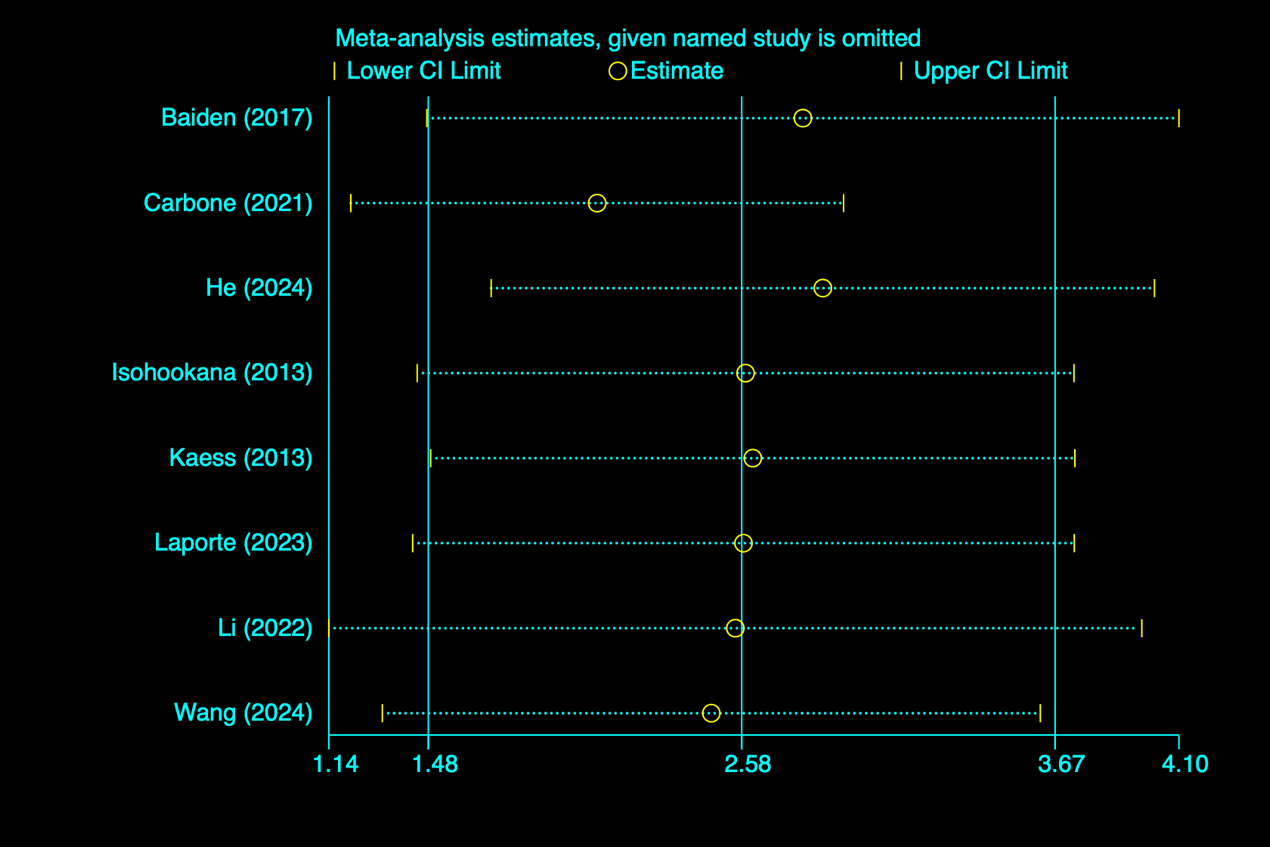


Figure S1 Sensitivity analysis of physical abuse


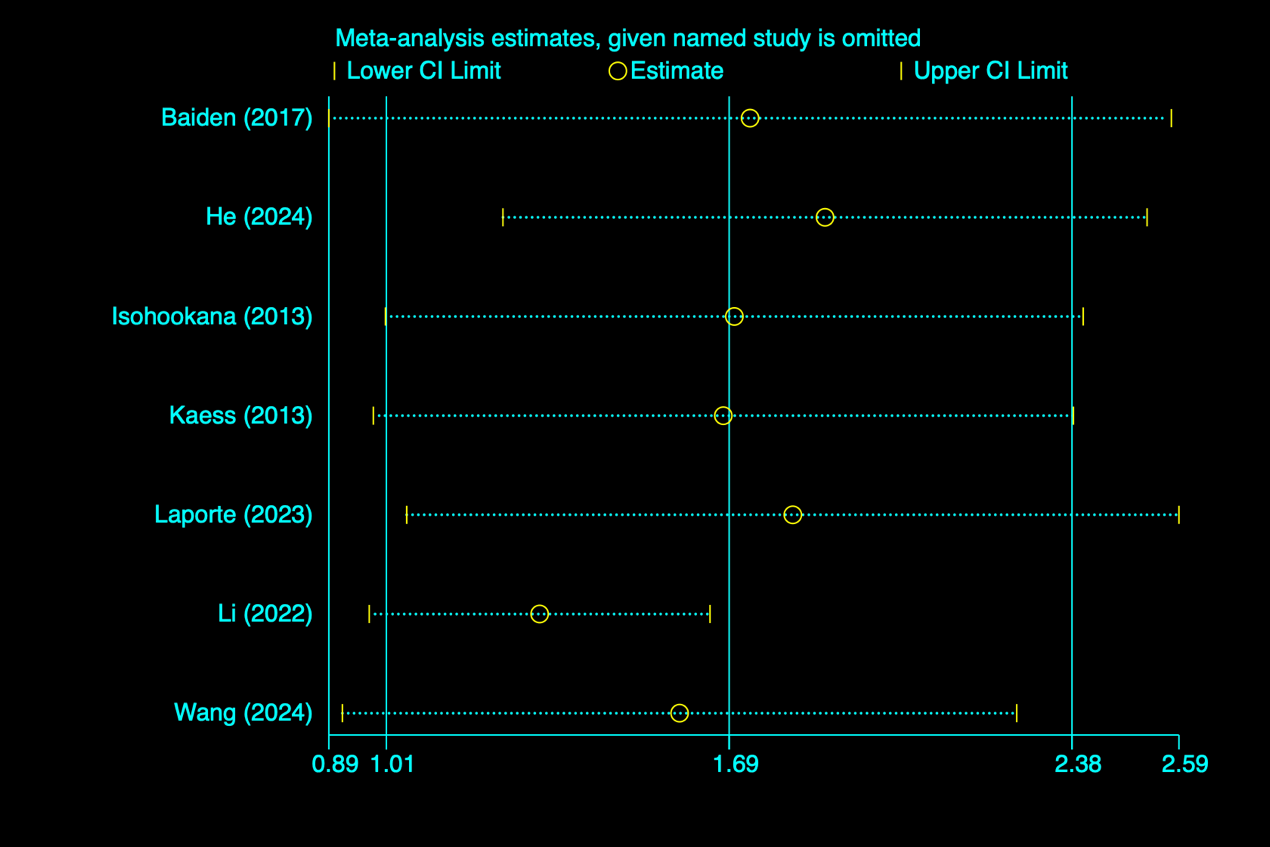


Figure S2 Sensitivity analysis of sexual abuse


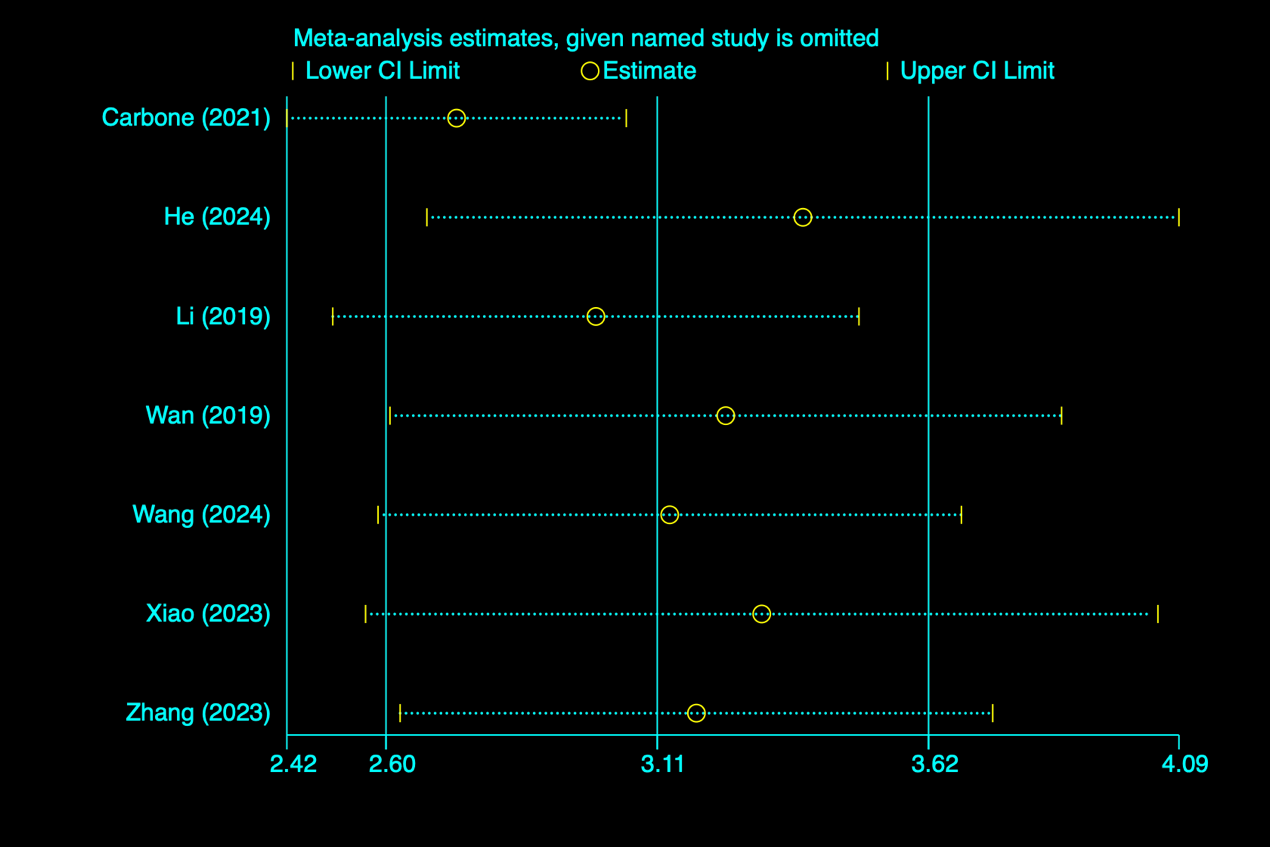


Figure S3 Sensitivity analysis of ACEs≥2


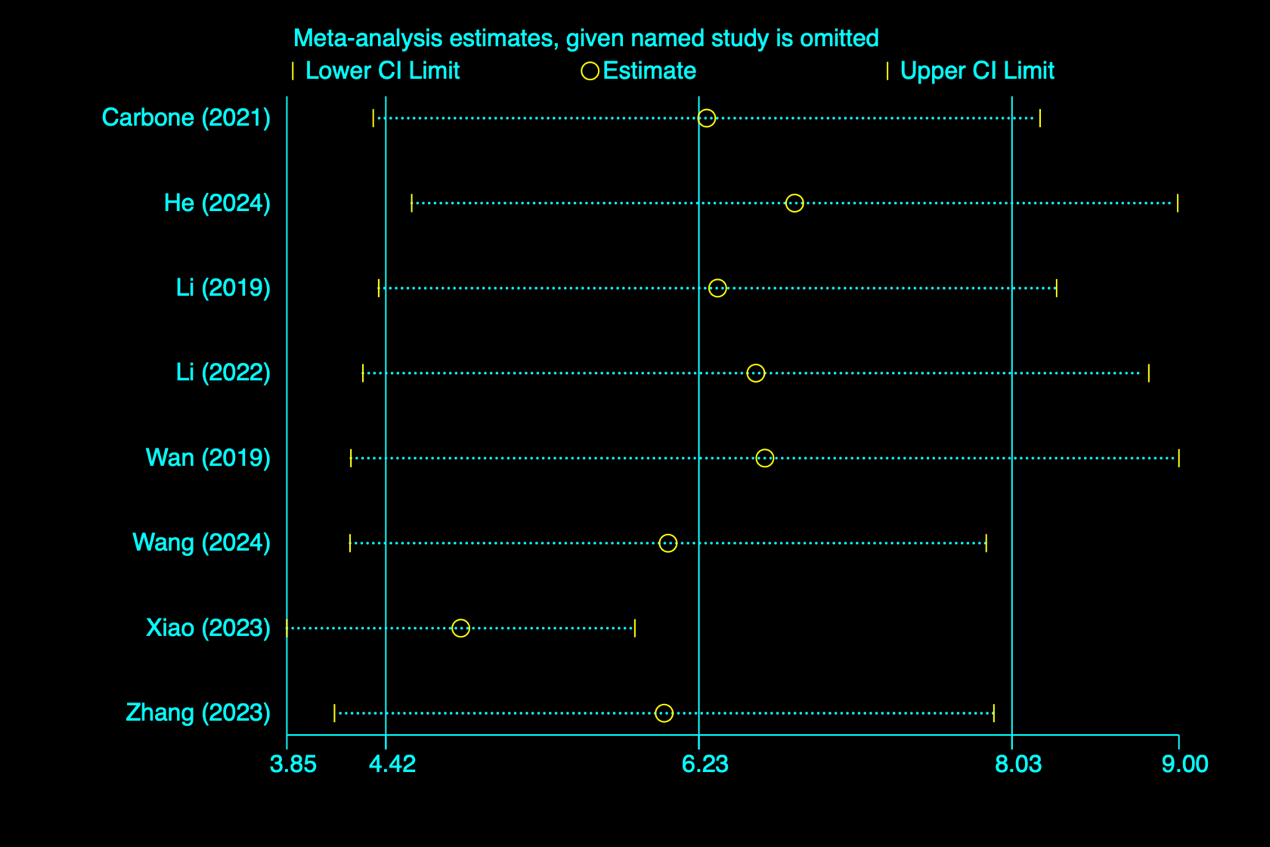


Figure S4 Sensitivity analysis of ACEs≥3


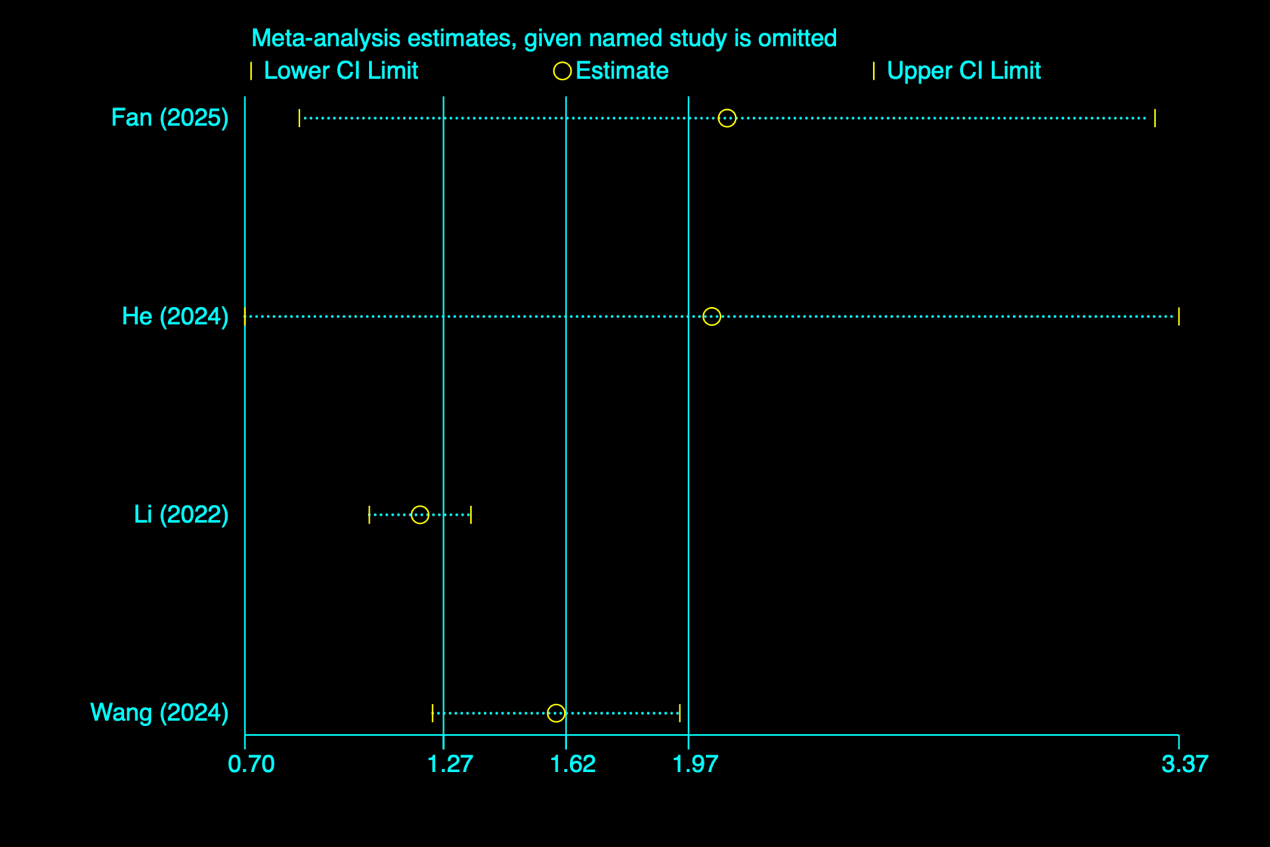


Figure S5 Sensitivity analysis of emotional abuse


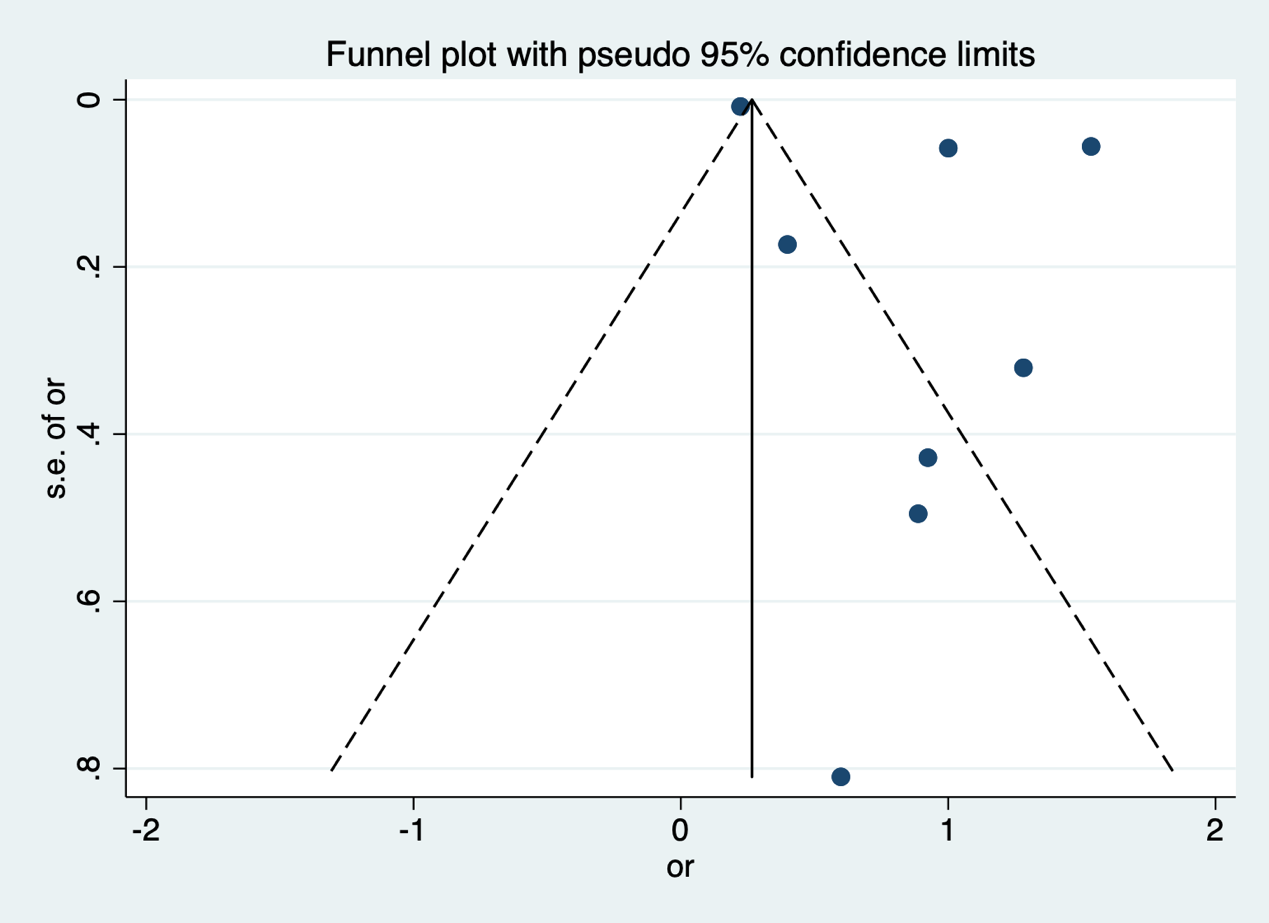


Figure S6 Funnel plot of Physical abuse


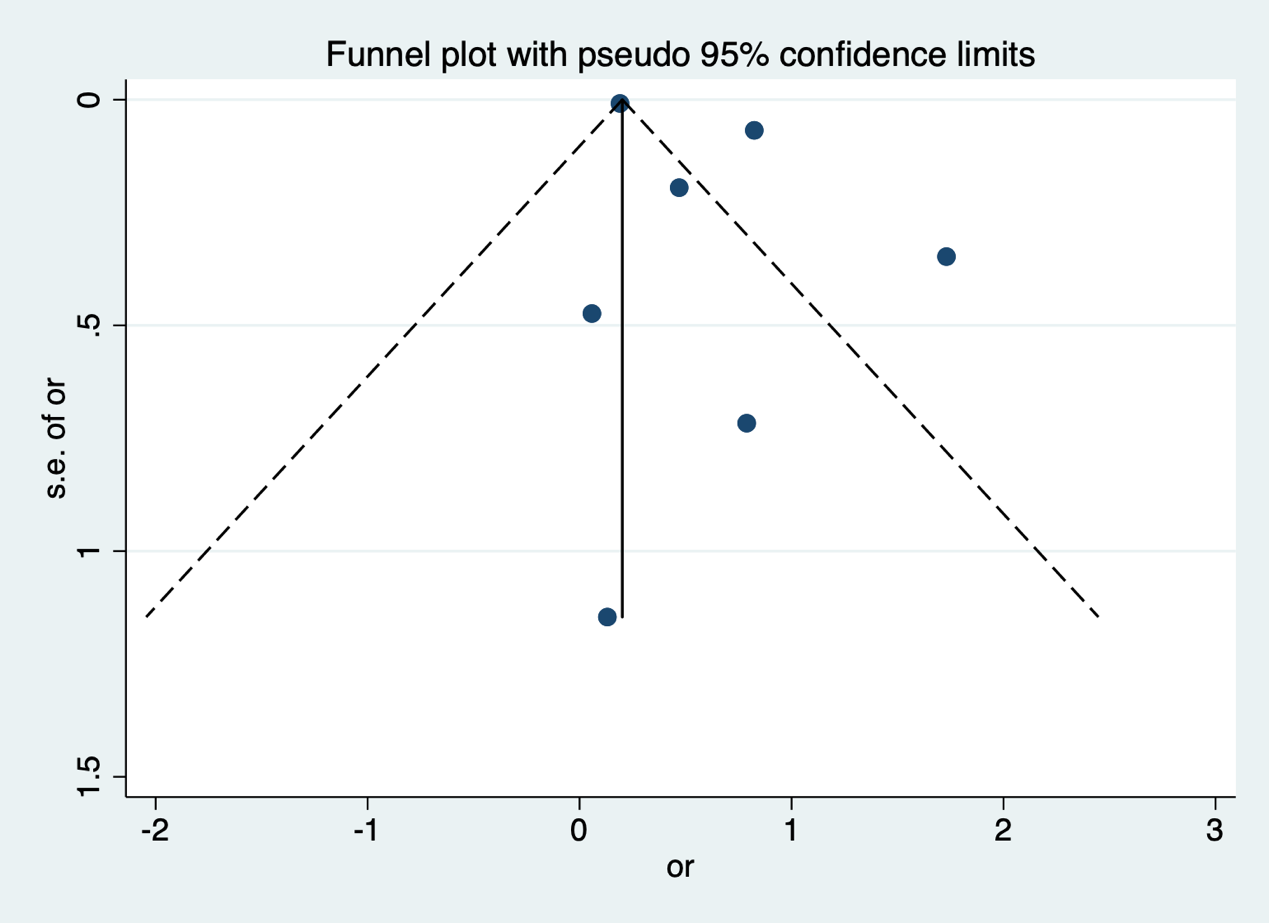


Figure S7 Funnel plot of Sexual abuse


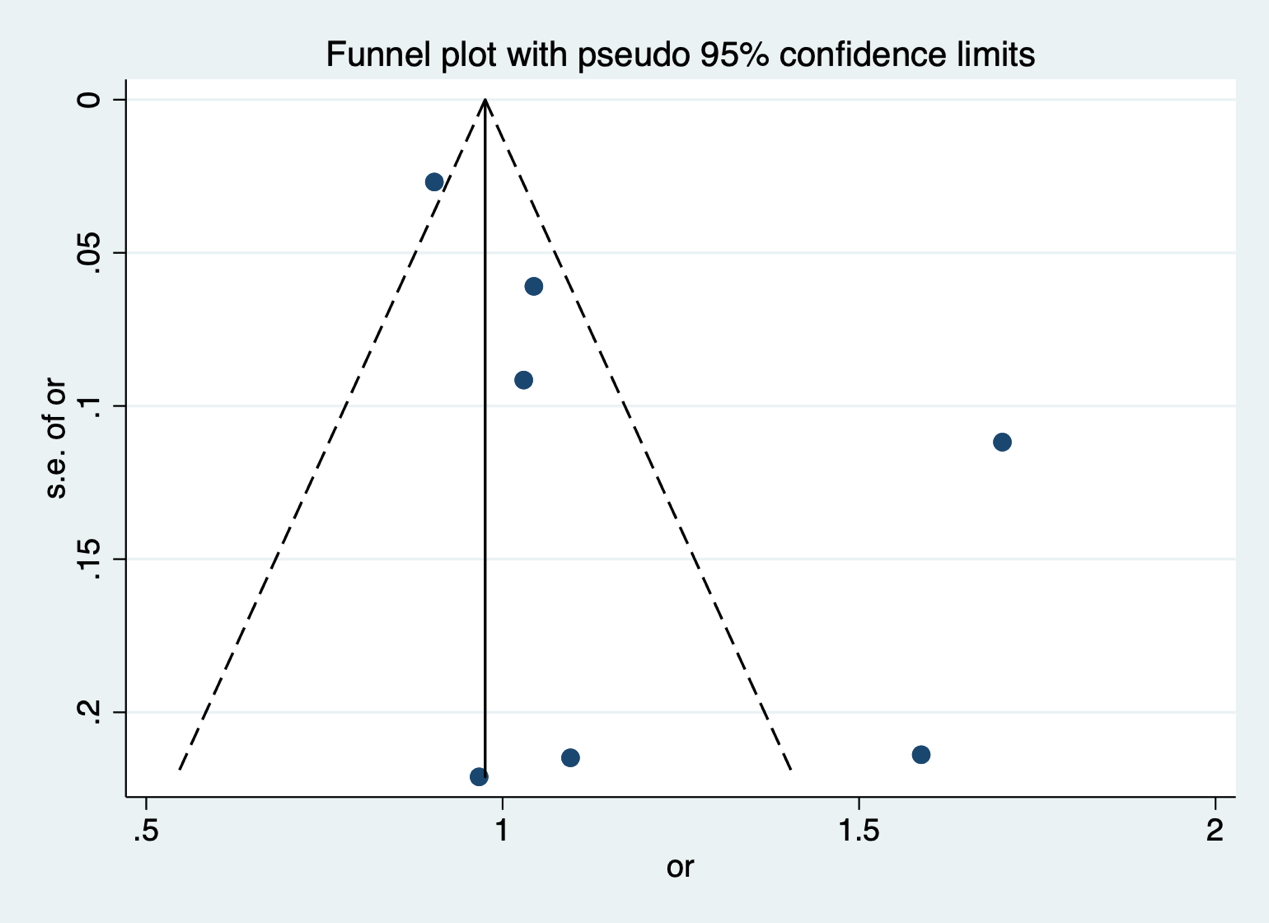


Figure S8 Funnel plot of ACEs≥2


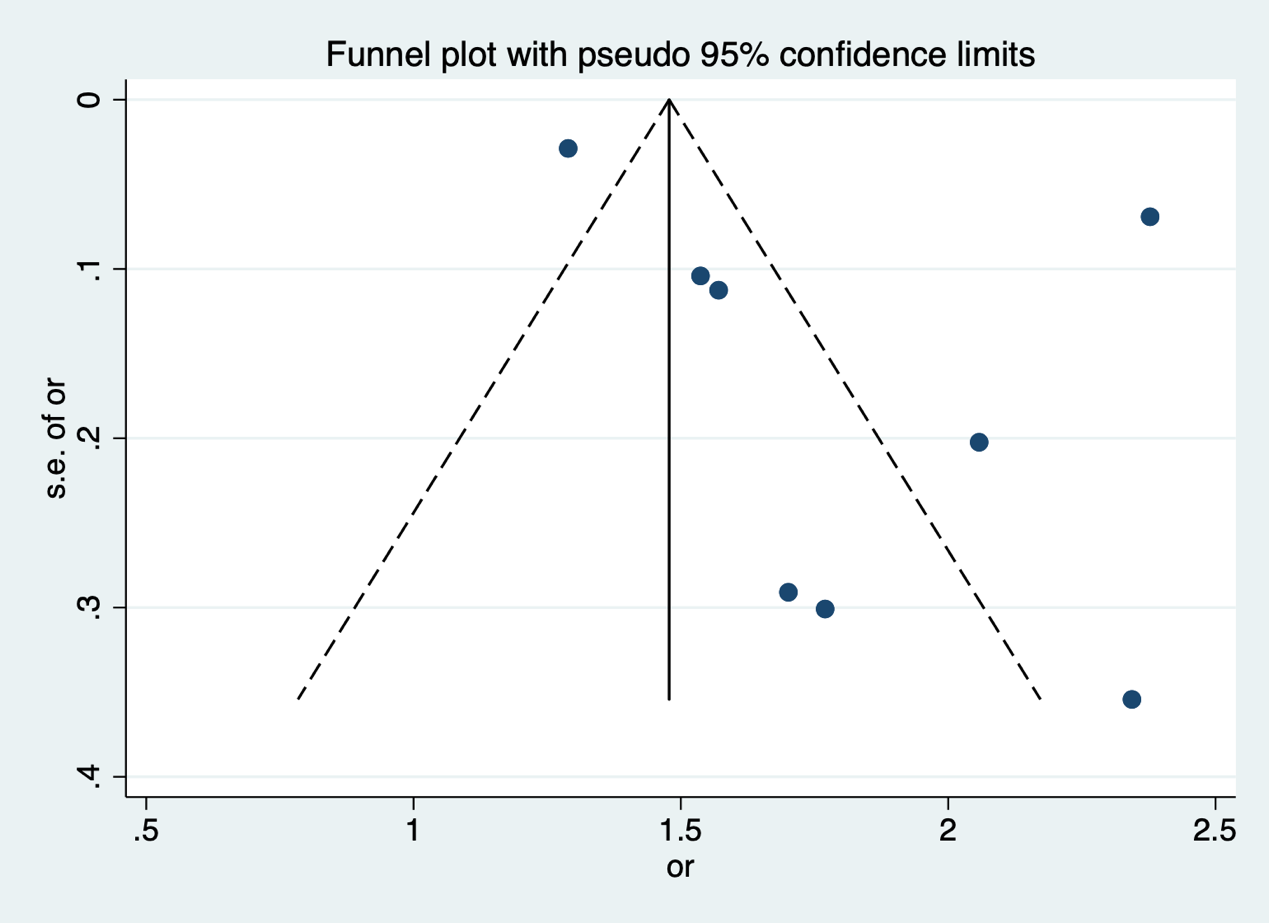


Figure S9 Funnel plot of ACEs ≥3


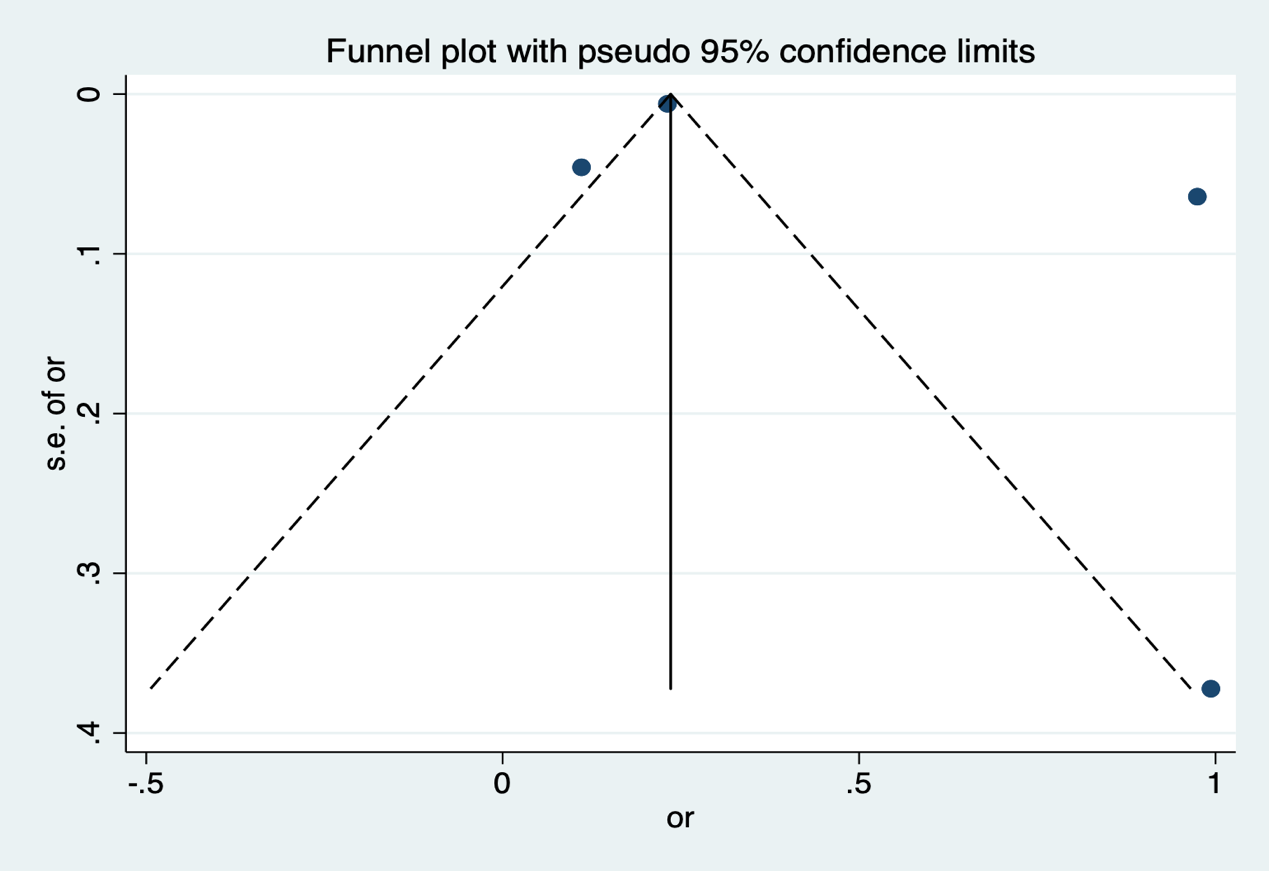


Figure S10 Funnel plot of Emotional abuse
